# Prediction models for breast cancer-related lymphedema: a systematic review and critical appraisal

**DOI:** 10.1186/s13643-022-02084-2

**Published:** 2022-10-13

**Authors:** Qiu Lin, Tong Yang, Jin Yongmei, Ye Mao Die

**Affiliations:** 1 Department of Nursing, 7th Hospital Affiliated to Shanghai University of Traditional Chinese Medicine, Shanghai, China; 2 Department of Nail-Breast Hernia Surgery, 7th Hospital Affiliated to Shanghai University of Traditional Chinese Medicine, Shanghai, China

**Keywords:** Second lymphedema, Predictive model, Risk factors, Prevention, Systematic review

## Abstract

**Purpose:**

The development of risk prediction models for breast cancer lymphedema is increasing, but few studies focus on the quality of the model and its application. Therefore, this study aimed to systematically review and critically evaluate prediction models developed to predict breast cancer-related lymphedema.

**Methods:**

PubMed, Web of Science, Embase, MEDLINE, CNKI, Wang Fang DATA, Vip Database, and SinoMed were searched for studies published from 1 January 2000 to 1 June 2021. And it will be re-run before the final analysis. Two independent investigators will undertake the literature search and screening, and discrepancies will be resolved by another investigator. The Prediction model Risk Of Bias Assessment Tool will be used to assess the prediction models’ risk of bias and applicability.

**Results:**

Seventeen studies were included in the systematic review, including 7 counties, of which 6 were prospective studies, only 7 models were validation studies, and 4 models were externally validated. The area under the curve of 17 models was 0.680~0.908. All studies had a high risk of bias, primarily due to the participants, outcome, and analysis. The most common predictors included body mass index, radiotherapy, chemotherapy, and axillary lymph node dissection.

**Conclusions:**

The predictive factors’ strength, external validation, and clinical application of the breast cancer lymphedema risk prediction model still need further research. Healthcare workers should choose prediction models in clinical practice judiciously.

**Systematic review registration:**

PROSPERO CRD42021258832

**Supplementary Information:**

The online version contains supplementary material available at 10.1186/s13643-022-02084-2.

## Background

In 2020, about 2.3 million new cases of female breast cancer were diagnosed and have surpassed lung cancer as the most commonly diagnosed in the world [1, 2]. Breast cancer-related lymphedema (BCRL) is a chronic complication that occurs after treatment for breast cancer, which can persist for a long-term and vicious circle. The incidence varies according to different study designs or timing, method of assessment, and relevant literature reports, and the incidence of BCRL is about 5–75.4% [3, 4]. The upper limb lymphedema can not only affect patients’ psychology with morphological changes, but accompanied by a series of symptoms can also bring life and work problems to patients [5]. At present, the treatment of chronic lymphedema is mainly to relieve symptoms, and the effect is not durable. Multiple studies have found that early detection and treatment of BCRL can prevent its progression and decrease the need for costly treatments [6, 7]. Therefore, there is a growing urgency to recognize and prevent BCRL early.

BCRL has different aspect risk factors, including demographic, physiological, biochemical, and treatment-related factors [8, 9]. In most of the studies, age is one of the risk factors for BCRL [10], but the predictor of age is not included in the BCRL prediction model in the study of prediction models commonly [11–14]. It may be related to the age of each included population and the different age stratification of each study. The impact of body mass index on BCRL has been confirmed in various studies, such as risk factors, model studies, and meta-analyses. Related studies have found that the occurrence of BCRL is closely related to serum phospholipid fatty acid composition and phenotype [15, 16]. Axillary radiotherapy and axillary lymph node dissection are considered to be the most important risk factors for lymphedema resulting from disruption of the lymphatic system [17, 18]. The effect of chemotherapy on BCRL is controversial. Norman et al. [19] found the lowest incidence occurs after SLNB and no chemotherapy. But the discussion in the study of Tsai et al. [20] shows that chemotherapy was not the direct cause of BCRL, and breast cancer patients receiving chemotherapy were more likely to receive invasive surgery and postoperative radiotherapy. Other risk factors such as edema within 3 months, lymphatic obstruction, inflammation, immune response, complement activation, wound healing, and fibrosis will affect the occurrence and development of lymphedema [21, 22]. In addition, there are many controversial risk factors and different research results.

A prediction model is a formal combination of multiple predictors. It calculates specific risk values for individuals so that it can predict the risk of related outcomes with intuitive data through quantitative methods [23]. It is a powerful tool for individualized diagnosis and treatment. In recent years, the development of the BCRL prediction model has gradually increased, but the study quality and results are different. Therefore, we aimed to systematically review and critically appraise all current prediction models for BCRL and provide a reference for clinical practice and future research.

## Materials and methods

All steps of this study were carried out according to the guide to systematic review and meta-analysis of the prediction model [24]. This systematic review was conducted and reported according to the Preferred Reporting Items for Systematic Reviews and Meta-Analyses (PRISMA) [25, 26] and registered on the International Prospective Register of Systematic Reviews PROSPERO (CRD42021258832), and the difference with registration is we added the Embase database.

### Information sources and search strategy

PubMed, Web of Science, Embase, MEDLINE, CNKI, Wang Fang DATA, Vip Database, and SinoMed were searched for studies published on 1 January 2000 and updated on 1 June 2021 in English and Chinese. We combined the following search terms which were used in referring to the PICOTS framework: the population (Breast Cancer), exposure (Lymphedema), and intervention (prediction model). Keywords were adjusted across databases. More details of the search strategy are given in the supplemental file. And it will be re-run before the final analysis.

### Selection criteria and data extraction

The review question was defined according to the PICOTS framework (see Table 1). In brief, any studies of a prediction model to predict the risk of the second lymphedema were included. Exclusion criteria were (1) only studied independent risk factors, (2) informal publication, and (3) systematic reviews or meta-analyses. Study screening mainly includes three steps. Firstly, the retrieval is imported into EndNotesX9 for duplicate data deletion, further screening through titles and abstracts (step 2), reading the full text (step 3), and screening the literature according to the inclusion and exclusion criteria by two independent reviewers (QL, YMD). And any conflicts are resolved by an independent reviewer (TY).

**Table 1:** Key items for framing the aim, search strategy, and study inclusion and exclusion criteria for systematic review

Item	Definition
Population	Breast cancer
Intervention	Any prediction model to predict lymphedema in breast cancer patients
Comparator	Not applicable
Timing	Predictive variables measured at any timepoint after surgery
Setting	Patients after breast cancer-related surgery in the ward or community

Data extraction will be conducted using a standardized data extraction form by two independent reviewers (QL, YMD) based on the recommendations in the Checklist for critical Appraisal and data extraction for systematic Reviews of prediction Modelling Studies (CHARMS). Any disagreement between reviewers was resolved by consensus. The key items to be extracted from each included study are 11 domains, including the source of data, participants, outcome(s) to be predicted, candidate predictors, sample size, missing data, model development, model performance, model evaluation, results, interpretation, and discussion; this information can be used to describe or assess the risk of bias or applicability. In addition, we extracted the general characteristics of the included studies, including author, publication year, and presentation of the model.

### Assessment of risk of bias and applicability

The bias risk and applicability of the study are evaluated by the bias risk assessment tool of the prediction model (PROBAST) [27]. The risk assessment of bias includes 20 questions in four domains: participants—concerned with the potential sources of bias and applicability related to the data sources used and participants’ selected, predictor—concerned with the potential sources of bias and applicability related to the definition and measurement of the predictors, outcome—concerned with the potential sources of bias and applicability related to the definition and determination of the outcome, and analysis—covers potential sources of bias and applicability concerns related to the analysis methods or statistical considerations. The answer to each question can be “yes,” “probably yes,” “probably no,” “no,” or “no information.” If a question has “no” or “probably no,” the risk of bias in related fields is high, and there is a high risk of bias in any field, then the overall risk of bias is high. Applicability assessment involved three domains, participants, predictors, and outcome. Each question was answered as “low concern regarding applicability,” “high concern regarding applicability,” and “unclear concern regarding applicability.” The domain of analysis is only assessed for risk of bias, having no applicability section [27].

## Results

### Study selection

**Fig. 1:**
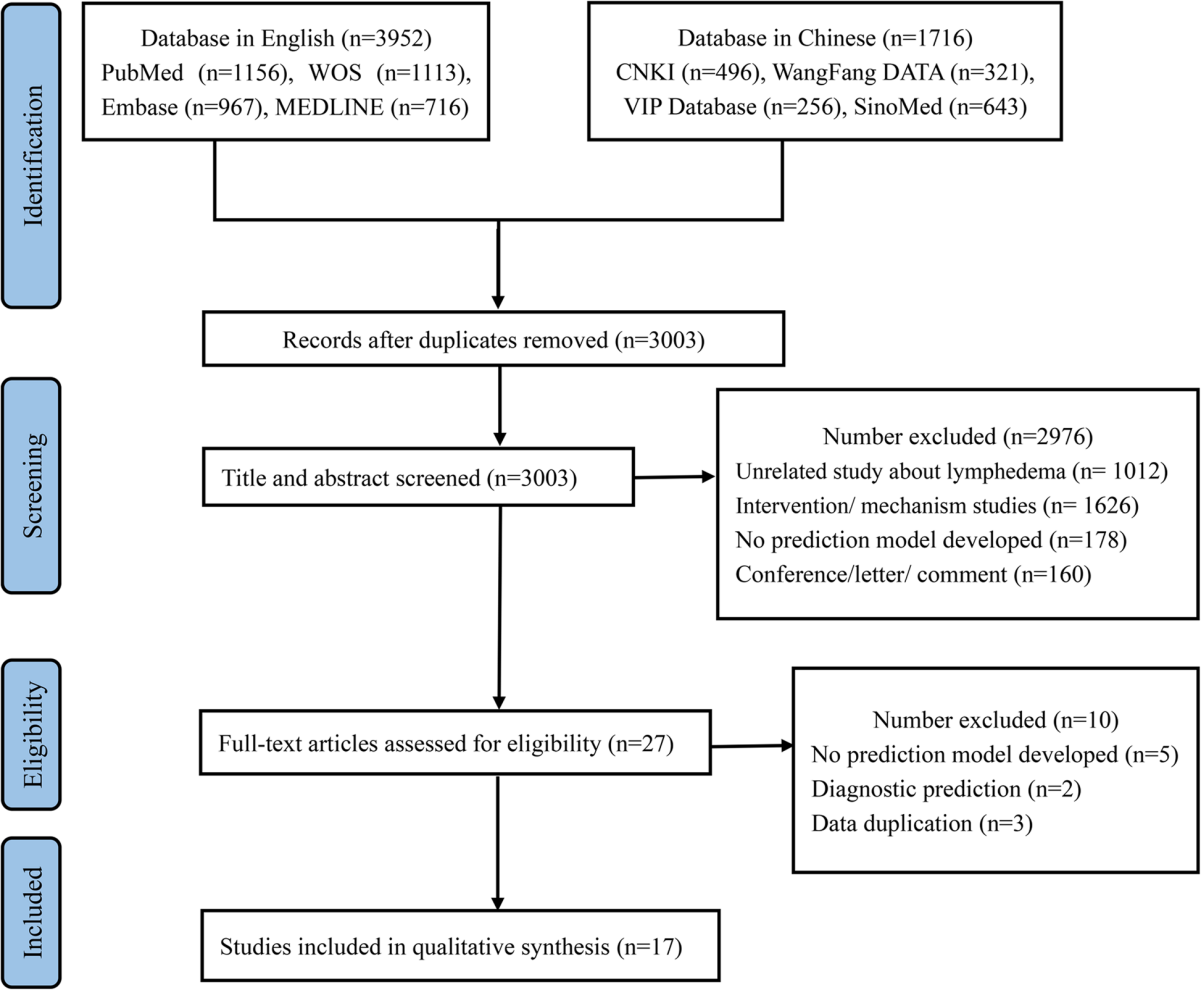
Identification of studies for the systematic review

The study retrieved 5668 titles through a systematic search. After the title and abstract were screened, twenty-seven studies were retained for full test assessment and 17 prediction models were ultimately selected for inclusion (see Fig. 1).

### Study characteristics

The study included 19,224 breast cancer patients from 7 countries (see Table 2). Most of BCRL prediction models were based on Chinese people (*n*=9, 52.94%) and 12 studies were published in the past 5 years. Most studies included various factors to establish the prediction model of breast cancer lymphedema: Yuan et al.’s study [14] was based on the normal level of lymphatic vessels, and Penn et al. [32] studied the prediction model of persistent lymphedema. Wei et al. [28] used symptoms as predictors to develop a risk prediction model for the early detection of lymphedema. Of the 17 studies, most were prospective cohorts (*n*=6, 35.29%), six were retrospective cohort studies, four were cross-sectional studies, and one was a randomized controlled trial.

**Table 2:** Characteristics of included studies

Year	First author	Country	Sample size(T/V)	Design	Outcome measure
2021	Wei X [28]	China	252/none	Cross-sectional	CD
2021	Yuan Q [14]	China	320/221	Prospective cohort	VD
2021	Liu Y F [12]	China	775/314	Retrospective cohort	Norman questionnaire
2021	Byun H K [29]	Korea	5549/1877 and 191	Retrospective cohort	CD
2021	Martinez J P [13]	Barcelona	504/none	Retrospective cohort	CD
2020	Li F L [30]	China	355/178	Case–control	CD
2020	Kwan J Y Y [31]	Canada	373/126	Prospective cohort	VD
2019	Gross J P [11]	Chicago	785/417	Randomized clinical trial	NI
2019	Penn I W [32]	China	342/none	Retrospective cohort	CD
2019	Yang X [33]	China	383/none	Retrospective cohort	CD
2018	Fu M R [34]	USA	355/none	Cross-sectional	Questions
2017	Basta M N [35]	USA	3136/none	Retrospective cohort	NI
2016	Wang L [36]	China	378/none	Prospective cohort	CD
2016	Dou W J [37]	China	221/none	Prospective cohort	CD
2014	Li H P [38]	China	346/none	Case–control	CD
2013	Kim M [39]	Korea	772/none	Prospective cohort	CD
2012	Bevilacqua J L [40]	Brazil	1054/none	Prospective cohort	VD

*T* Train cohort, *V* Validation cohort, *CD* Circumferent difference, *VD* Volumetric difference, *NI* No information

There are different outcome diagnostic criteria for each model study. Ten studies were evaluated by the perimeter measurement; Penn et al. [32] set the boundary value at 1 cm and diagnosed combined with symptoms of BCRL. Although the measurement positions of other researchers were different, they all set the boundary value at 2 cm. Kim et al. [39] diagnosed lymphedema with swelling of the affected arm exceeding 5% of the circumference difference; 5 studies used the capacity difference to diagnose BCRL, which also had a threshold difference. Li and Bevilacqua et al. [30, 40] indicated that the volume difference between the arms was greater than 200 mL, and Yuan et al. [14] indicated that the volume difference was more than 10%, which was diagnosed as lymphedema.

### Development and validation of the model

The modeling method of BCRL prediction models used mostly was logistic regression analysis (*n*=10, 58.82%). Other studies were five Cox models, one used linear regression, and one used machine learning to estimate the probability of lymphedema (see Table 3). The candidate variables of each study can be divided into treatment-related, self-related, and behavior variables. Most of the studies focus on the treatment methods of diseases and the physiological conditions of patients. Compared with foreign countries, Chinese researchers focus on the influence of patients’ behavior on lymphedema. For example, Liu et al. and Li et al. [12, 38] include the exercise of the affected arm and the level of physical activity. The predictors included from 3 to 7 in the study. The most common risk factors included were BMI, radiotherapy, chemotherapy, and axillary lymph node dissection.

**Table 3:** Characteristics of studies included in the development and validation of the model

Author	Modeling method	Predictors in the final model	Model performance		Model presentation	Median follow-up time	Internal validation	External validation
			Discrimination (I/E)	Calibration				
Wei X	Logistic regression	17 symptoms	ED: AUC = 0.889 (0.840–0.938) LD: AUC = 0.925 (0.883–0.967)	Calibration plot ED: Brier scores = 0.141 LD: Brier scores = 0.098	Unclear	None	10-fold cross-validation	None
Yuan	Logistic regression	Proportion of the arm lymph above the level of axillary vein/BMI/radiotherapy/chemotherapy	AUC 0.829/0.804	Calibration plot, Hosmer–Lemeshow test	Nomogram	29 months	Bootstrap	Geographical
Liu Y F	Logistic regression	Modified radical mastectomy/postsurgical infection/chemotherapy/radiotherapy/exercise of affected arm/level of physical activity per week/BMI	AUC 0.721/0.702	Calibration plot discrimination	Nomogram	42 months	None	Geographical
Hwa Kyung Byun	Cox hazard model	BMI, greater number of dissected/positive nodes, use of chemotherapy/extent of surgery/fractionation/field of RT	C-index and AUC I: 0.774 and 0.750; E: Set1: 0.832 and 0.782; Set2: 0.820 and 0.802	Calibration plots	Nomogram	60 months	Bootstrap	Geographical
Martinez J P	Logistic regression	BMI/postoperative complications/no. of lymph nodes extracted/level of lymph node dissection/lymph node status	AUC 0.68	None	None	2 years	10-fold cross-validation	None
Li F L	Logistic regression	Type of surgery/type of axillary lymph node surgery/early edema on affected arm/neoadjuvant chemotherapy/radiotherapy/use the affected arm to lift or carry heavy objects suddenly	AUC 0.736	Hosmer–Lemeshow test	Scoring system	None	None	None
Kwan J Y Y	Linear regression	Age/BMI/mammographic breast density/number of pathological lymph nodes/axillary lymph node dissection	AUC Mild: 0.72 Severe: 0.83	None	Equation	1.1years	2:1 split.	None
Gross J P	Logistic regression	BMI/nodes removed/RNI field group	C-index 0.96/0.71	Calibration plot	Nomogram	None	Bootstrap	Temporal
Penn I W	Logistic regression	Lymph node metastases/CD	AUC 0.908/-	None	None	5 years	None	None
Yang X	Cox hazard model	Radiotherapy/weight increase/no. of dissected axillary nodes/knowledge of lymphedema prevent	C-index Model A: 0.77; model B: 0.73	Hosmer–Lemeshow test	Nomogram	3 years	None	None
Mei R. Fu	Machine learning	Symptoms, individual personal and clinical characteristics, quality of life, self-care strategies	AUC 0.751	None	None	None	None	None
Basta M N	Cox hazard model	Age/BMI/invasive cancer diagnosis/postmastectomy radiation/axillary dissection	C-index 0.77	Hosmer–Lemeshow statistic	Table	4.2 years	None	None
Wang L	Logistic regression	Hypertension/surgery on dominant arm/level of ALND/radiotherapy/surgical infection/seroma/early edema	AUC 0.798	Calibration plot	Additive scoring system	12 months	Bootstrap	None
Dou W J	Logistic regression	Level of lymph node dissection/radiotherapy/functional exercise/avoiding strenuous exercise/not lifting weight	AUC 0.815	Hosmer–Lemeshow statistic	Nomogram	1 year	None	None
Li H P	Logistic regression	Tumor site/type of surgical incision/level of axillary lymph node dissection/radiotherapy/no neglect of upper limb or chest edema/avoiding strenuous exercise/avoiding injury	AUC Logistic scoring system: 0.836; additive scoring system: 0.834	Hosmer–Lemeshow statistic	Additive scoring system	None	None	None
Kim M	Cox hazard model	Pathologic stage/no. of dissected axillary nodes/adjuvant chemotherapy/radiation therapy	None	None	None	5.1 years	None	None
Bevilacqua J L	Cox hazard model	Model 1: age/BMI/neoadjuvant chemotherapy Model 2: age/BMI/neoadjuvant chemotherapy/level of axillary lymph node dissection/radiotherapy Model 3: age/BMI/neoadjuvant chemotherapy/level of axillary lymph node dissection/radiotherapy/seroma/early edema	AUC Model 1:0.706 Model 2: 0.729 Model 3: 0.736	None	Nomogram	41 months	Bootstrap	None

*BMI* Body mass index, *ED* Early detection, *LD* Late detection, *AUC* Area under the curve, *I* Internal validation, *E* External validation, *RT* Radiation therapy, *ALND* Axillary lymph node dissection, *RNI* Regional nodal irradiation, *CD* Circumferent difference

Of all models included, only 10 (58.82%) models were internally validated, including 6 (35.29%) bootstrap validation, 2 (12.5%) random split-sample validation, and 2 cross-validation. Four (23.53%) models were externally validated. Most studies assessed discrimination with concordance statistics (c-statistic) or the receiver operating characteristic (ROC) curve, and the ROC was 0.68~0.96 in the training cohort and 0.702~0.804 in the validation cohort. Only 11 (64.70 %) reported calibration, 5 studies assessed calibration with Hosmer–Lemeshow tests, and only the study of Wei et al. [28] assessed calibration with Brier scores to quantify calibration.

### Risk of bias and applicability

**Fig. 2:**
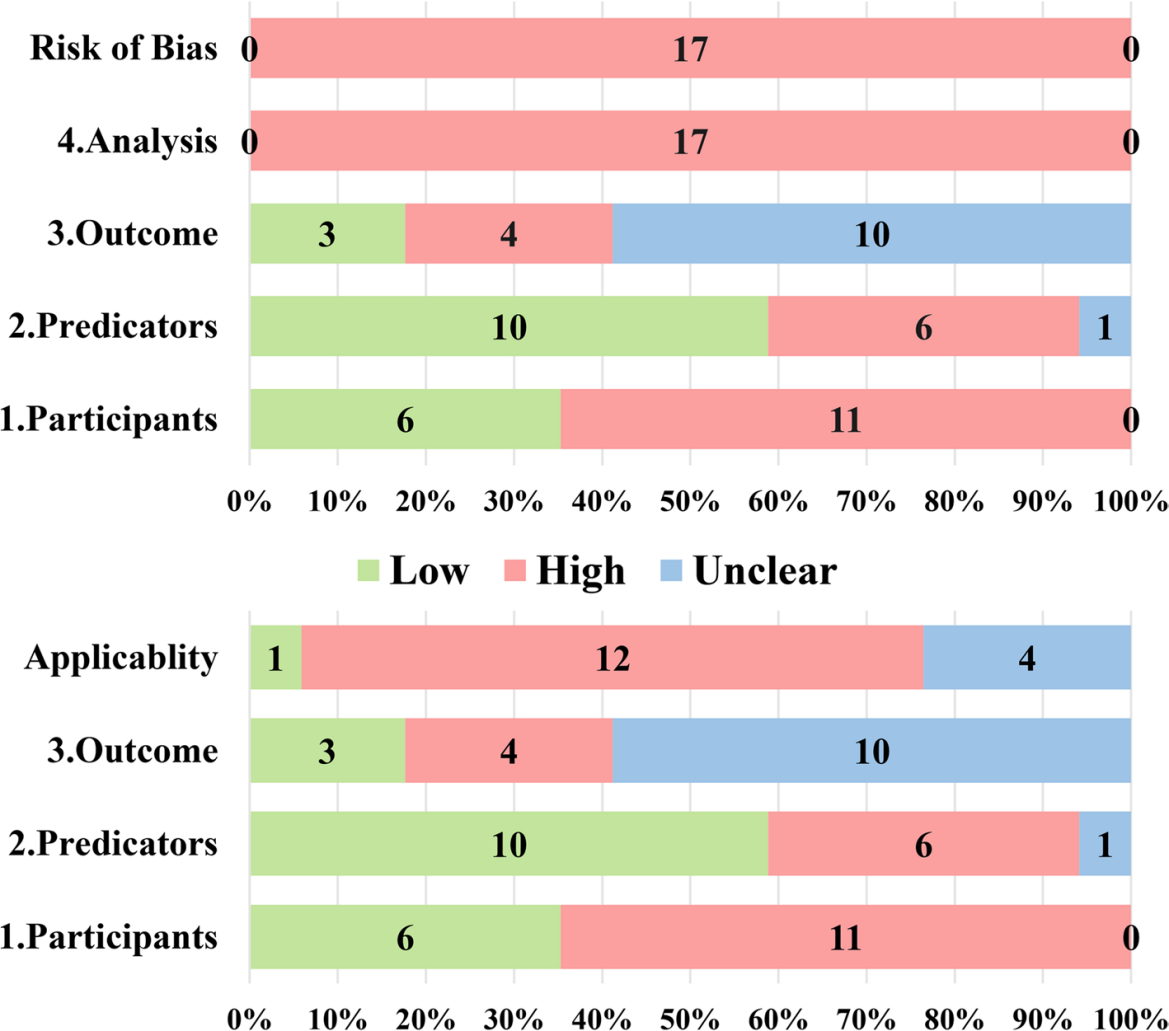
Risk of bias and applicability assessment

More than half of the studies were at high risk of bias principally due to issues in the participants’ domain and analysis domain. The overall and domain-specific ratings for risk of bias and applicability are reported in Fig. 2.

### Domain 1: Participants

Although most of the data sources of the study were cohort studies (*n*=12, 70.59%), there is a high risk in the bias risk of the criteria for participant selection (see Fig. 2). For example, the study by Liu et al. [12] limited population and included those who had completed breast cancer surgery at least 6 months, and patients who had not been included in the study within 6 months after surgery probably cause selection bias. The study by Gross et al. [11] used a data derived from randomized controlled trials. The participants were only included if they received radiotherapy, and those who did not receive radiotherapy were not included in the study.

### Domain 2: Predictors

Bias in the predictors’ domain mostly was a low concern for risk (*n*=10, 58.82%). For the risk of bias caused by different definitions and measurements of the predictors, the source data are mainly multi-center study data, and the model included in this study has no multi-center research data, and some studies have clarified the definition of relevant predictors. In this domain, there is a high risk of bias from the evaluation of predictive factors about knowledge of outcome data, and there is no “blinding” for predictor assessments [27, 41, 42]. The study by Yang et al. [33] is a retrospective cohort study, and it is not known whether the researchers evaluate the predictive factors in the case of unclear patient outcome data. However, the assessment of patients with lymphedema probably has bias. Kwan et al. [31] conducted a prospective cohort study and did not have an outcome in advance, and there was no risk of bias in the assessment of predictors.

### Domain 3: Outcome

Of all studies, most were unclear for risk in the outcome domain (*n*=10, 58.82%). The unclear items mainly focus on the researchers who are unclear about the information of predictors and whether the interval between predictor assessment and outcome determination is appropriate. The included studies do not mention these two parts. Knowledge of predictor results may influence determination and lead to bias [27, 43, 44]. Most of the studies (*n*=10, 58.82%) used the circumference difference to diagnose lymphedema, which was a semi-objective indicator. There is no clear definition of the follow-up interval and the time to determine the outcome. The follow-up time of Penn et al. [32] was every 3 months in the first 2 years after surgery and every 6 months in the third to fifth years and once per year after that. According to related research, the follow-up interval and the time to determine the outcome is appropriate.

### Domain 4: Analysis

All studies received a high concern for risk in the analysis domain. Nine signaling questions facilitate the risk of bias judgment for this domain. The risk of bias was mainly derived from questions 1, 4, 5, 6, and 8, of which 1 was the sample size, and the researchers believed the number of events per variable (EPV) should be at least 20 [45, 46]. And in Liu et al., Kim et al., Wang et al., Penn et al., Basta et al., Byun et al., and Martinez-Jaimez et al. [13, 29, 30, 32, 35, 36, 39], EPV was higher than 20. Question 4 is about participants with missing data handled: most studies did not report changes in follow-up data; only Yuan et al., Liu et al., and Li et al. [12, 14, 30] were at a low-bias risk; the study of Yuan et al. [14] has no missing data; and Liu et al. and Li et al. [12, 30] are cross-sectional analysis without missing data. Question 5 is about the selection of predictors, and only Gross et al. and Martinez-Jaimez et al. [11, 13] avoided selecting the predictors with a statistically significant univariable association. Question 8 is about the model performance. Most studies have internally validated the use of the training data indirectly, while Li et al. and Kwan et al. [30, 31] were only randomly split-sample for validation.

### Applicability

The applicability of the study is assessed for the participants, predictors, and outcome domains. Only one of the models included in this study is of low concern regarding applicability. The models established by Yuan et al., Wang et al., Kwan et al., and Basta et al. [14, 31, 35, 36] were of unclear concerns regarding applicability and Kim et al. [39] were of low concerns regarding applicability. The remaining 12 studies were of high concern regarding applicability.

## Discussion

In this systematic review of BCRL prediction models, we identified 17 model development studies. The development of the prediction model included was deemed to be at a high risk of bias owing to a combination of poor reporting and methodological conduct for participant selection, predictor description, and statistical methods used, but most models reported moderate to excellent predictive performance.

Several aspects could influence the occurrence of lymphedema for breast cancer. Due to the different populations, candidate predictors, and modeling methods in the primary studies, the final predictors are included in prediction models. More importantly, the methods for handling continuous and categorical predictors included in each study are different. For the level of lymph node dissection, most are divided into I~III; this classification is rarely applied in clinical practice at present. There are studies on the number of lymph node dissection stratification, but the number is very different. For example, Kim et al.’s study [39] is bounded by the number of dissections 10, and Yang et al.’s study [33] is leveled by the number of dissections 7 and 15. For radiotherapy, it can be divided into whether radiotherapy and radiotherapy area, the study of Yuan et al., Liu et al., and Gross et al. [11, 12, 14]. BMI was presented as a continuous variable, while Yang et al. [33] classified it by 18.5 and 22.9. However, most of the prediction models included easy-to-measure predictors, enhancing their applicability to clinic practice and self-management of breast cancer patients.

In recent years, there are more and more researches on predictive models in medicine [47]. However, few were validated in external populations. In our study, there are 12 BCRL prediction models in the past 5 years [11–14, 28–35], which indicate that the research on this risk prediction model is still in the progress stage. The study of the prediction model includes a search for prognostic and diagnostic factors, research on the development of the prediction model without external validation, the development of the prediction model with external validation, validation study of the prediction model, and the influence of the prediction model. External validation uses independent data to evaluate the calibration and discrimination of the model, which can include external verification types of a different time, different space, and different scenarios [48, 49]. Due to time, resources, and other reasons, researchers generally cannot access multiple data, and external validation might be limited. And data sharing is proposed to offer the possibility of making full use of all available data [50]. The model developed by Bevilacqua is a widely used prediction model, Du et al. [51] validated it by applying to 203 breast cancer patients for retrospective analysis. The results showed that the AUC value was 0.711, indicating that the model had a good discrimination ability.

The external validation of the model by different authors is one of the methods to promote the application of the model in clinical practice, but the operability of the model is worth thinking about. The comprehensive report of the parameters of the model is the primary condition for other researchers to use the existing model, and the TRIPOD statement also has corresponding requirements for presenting important data so that the model can facilitate external validation by other researchers after publication [49], such as all regression coefficients and model intercept or baseline survival at a given time point. Gross et al. [11] directly present the nomogram, and the regression coefficient or the weight of the calculation method of risk score was not reported. The Cox model constructed by Li et al. [30] only reported the calculation method of risk score, model intercept, or basic survival probability. At present, the risk prediction model mainly includes a nomogram, equation, and table. Most of the prediction models in our study included are nomograms; the studies of Martinez-Jaimez et al., Penn et al., and Kim et al. [13, 32, 39] only report the final included predictors, without the specific content of the prediction model. In addition, the detailed description of the risk prediction model can promote its clinical application. Although researchers can determine the applicability of the model by reading the research methods of the model and the explanation of the included predictors, it is more important for potential users to visually present information.

### Strength and limitations

To our best knowledge, this study is the first to systematically review and appraise the prediction model for breast cancer-related lymphedema. And in our study, the research plan and information registration are carried out before the study, and the normative research and report are carried out through the Cochrane manual and CHARMS.

There are potential limitations to our study. First, due to the differences in diagnostic criteria, included predictive factors, modeling methods, and evaluation indexes, it is inappropriate to carry out the meta-analysis. Second, all model studies on BCRL prediction were included at different modeling methods, and PROBAST might not be suitable for the model evaluation of machine learning. Finally, we restricted our focus to Chinese- and English-related databases; there may be prediction models for other languages that are not examined here.

## Conclusions

Several prediction models for BCRL are currently available and they all report good discriminative performance. However, these models have a high risk of bias and lack external validation. Therefore, further studies aimed at validating models externally to evaluate the extrapolation of the model. And the development of prediction models is expected to improve the transparent reporting of the study, so that the model will facilitate external validation by other researchers and contribute to the clinical application after publication. Eligible prediction models will help to identify high-risk groups of breast cancer lymphedema early, can enhance patient care, and promote rational allocation of limited medical resources.

## Supplementary Information

**Additional file 1.** Database Search Strategy in English.

## Data Availability

We have control of all data and agree to allow the journal to review our data if required.

## Abbreviations

BCRL: Breast cancer-related lymphedema
PRISMA: Preferred Reporting Items for Systematic Reviews and Meta-Analyses
CHARMS: Checklist for critical Appraisal and data extraction for systematic Reviews of prediction Modelling Studies
PROBAST: Prediction model Risk Of Bias Assessment Tool
ROC: Receiver operating characteristic
AUC: Area under the curve
TRIPOD: Transparent Reporting of a multivariable prediction model for Individual Prognosis or Diagnosis
