# Supplementary material for: Prediction models for breast cancer-related lymphedema: a systematic review and critical appraisal
Source: Syst Rev. 2022 Oct 13;11:217. doi: 10.1186/s13643-022-02084-2 (PMC9559764; doi:10.1186/s13643-022-02084-2)
Supplement: Supplementary file 1 — Additional file 1. Database Search Strategy in English. [file 13643_2022_2084_MOESM1_ESM.docx]

**SUPPORTING INFORMATION**

**Additional file 1. Database Search Strategy in English.**

| #1 | "Breast Neoplasms"[MeSH Terms] OR "breast cancer"[Title] OR "breast tumor"[Title] OR "mammary neoplasm"[Title] OR "mammary carcinoma"[Title] OR "breast neoplasm"[Title] OR "breast carcinoma"[Title] OR "breast malignan*"[Title] OR "breast metastas*"[Title] OR "mammary malignan*"[Title] OR "mammary metastas*"[Title] | [346,712](https://pubmed.ncbi.nlm.nih.gov/?term=%22Breast+Neoplasms%22%5BMeSH+Terms%5D+OR+%22breast+cancer%22%5BTitle%5D+OR+%22breast+tumor%22%5BTitle%5D+OR+%22mammary+neoplasm%22%5BTitle%5D+OR+%22mammary+carcinoma%22%5BTitle%5D+OR+%22breast+neoplasm%22%5BTitle%5D+OR+%22breast+carcinoma%22%5BTitle%5D+OR+%22breast+malignan%2A%22%5BTitle%5D+OR+%22breast+metastas%2A%22%5BTitle%5D+OR+%22mammary+malignan%2A%22%5BTitle%5D+OR+%22mammary+metastas%2A%22%5BTitle%5D&sort=date&size=200&show_snippets=off) |
| --- | --- | --- |
| #2 | "Lymphedema"[MeSH Terms] OR "lymphoedema"[Title] OR "Lymphedema"[Title] OR "lymphedemas"[Title] OR "lymphatic edema"[Title] OR "oedema"[Title] OR "edema"[Title] OR "swelling"[Title] OR "elephantias*"[Title] Sort by: Most Recent | [56,455](https://pubmed.ncbi.nlm.nih.gov/?term=%22Lymphedema%22%5BMeSH+Terms%5D+OR+%22lymphoedema%22%5BTitle%5D+OR+%22Lymphedema%22%5BTitle%5D+OR+%22lymphedemas%22%5BTitle%5D+OR+%22lymphatic+edema%22%5BTitle%5D+OR+%22oedema%22%5BTitle%5D+OR+%22edema%22%5BTitle%5D+OR+%22swelling%22%5BTitle%5D+OR+%22elephantias%2A%22%5BTitle%5D%0D%0A&sort=date&size=200&show_snippets=off) |
| #3 | "Risk"[MeSH Terms] OR "risk*"[Title/Abstract] OR "risk factor"[Title/Abstract] OR "risk factors"[Title/Abstract] OR "age"[Title/Abstract] OR "BMI"[Title/Abstract] OR "modified radical mastectomy"[Title/Abstract] OR "infection"[Title/Abstract] OR "chemotherapy"[Title/Abstract] OR "Radiotherapy"[Title/Abstract] OR "physical activity"[Title/Abstract] OR "exercise"[Title/Abstract] OR "early edema"[Title/Abstract] OR "seroma"[Title/Abstract] OR "hypertension"[Title/Abstract] OR "behavior"[Title/Abstract] OR "prevention"[Title/Abstract] | [7,333,884](https://pubmed.ncbi.nlm.nih.gov/?term=%22Risk%22%5BMeSH+Terms%5D+OR+%22risk%2A%22%5BTitle%2FAbstract%5D+OR+%22risk+factor%22%5BTitle%2FAbstract%5D+OR+%22risk+factors%22%5BTitle%2FAbstract%5D+OR+%22age%22%5BTitle%2FAbstract%5D+OR+%22BMI%22%5BTitle%2FAbstract%5D+OR+%22modified+radical+mastectomy%22%5BTitle%2FAbstract%5D+OR+%22infection%22%5BTitle%2FAbstract%5D+OR+%22chemotherapy%22%5BTitle%2FAbstract%5D+OR+%22Radiotherapy%22%5BTitle%2FAbstract%5D+OR+%22physical+activity%22%5BTitle%2FAbstract%5D+OR+%22exercise%22%5BTitle%2FAbstract%5D+OR+%22early+edema%22%5BTitle%2FAbstract%5D+OR+%22seroma%22%5BTitle%2FAbstract%5D+OR+%22hypertension%22%5BTitle%2FAbstract%5D+OR+%22behavior%22%5BTitle%2FAbstract%5D+OR+%22prevention%22%5BTitle%2FAbstract%5D+&sort=date&size=200&show_snippets=off) |
| #4 | #1 AND #2 AND #3 AND (2000:2022[pdat]) | [1,156](https://pubmed.ncbi.nlm.nih.gov/?term=%28%28%22Risk%22%5BMeSH+Terms%5D+OR+%22risk%2A%22%5BTitle%2FAbstract%5D+OR+%22risk+factor%22%5BTitle%2FAbstract%5D+OR+%22risk+factors%22%5BTitle%2FAbstract%5D+OR+%22age%22%5BTitle%2FAbstract%5D+OR+%22BMI%22%5BTitle%2FAbstract%5D+OR+%22modified+radical+mastectomy%22%5BTitle%2FAbstract%5D+OR+%22infection%22%5BTitle%2FAbstract%5D+OR+%22chemotherapy%22%5BTitle%2FAbstract%5D+OR+%22Radiotherapy%22%5BTitle%2FAbstract%5D+OR+%22physical+activity%22%5BTitle%2FAbstract%5D+OR+%22exercise%22%5BTitle%2FAbstract%5D+OR+%22early+edema%22%5BTitle%2FAbstract%5D+OR+%22seroma%22%5BTitle%2FAbstract%5D+OR+%22hypertension%22%5BTitle%2FAbstract%5D+OR+%22behavior%22%5BTitle%2FAbstract%5D+OR+%22prevention%22%5BTitle%2FAbstract%5D%29+AND+%28%22Breast+Neoplasms%22%5BMeSH+Terms%5D+OR+%22breast+cancer%22%5BTitle%5D+OR+%22breast+tumor%22%5BTitle%5D+OR+%22mammary+neoplasm%22%5BTitle%5D+OR+%22mammary+carcinoma%22%5BTitle%5D+OR+%22breast+neoplasm%22%5BTitle%5D+OR+%22breast+carcinoma%22%5BTitle%5D+OR+%22breast+malignan%2A%22%5BTitle%5D+OR+%22breast+metastas%2A%22%5BTitle%5D+OR+%22mammary+malignan%2A%22%5BTitle%5D+OR+%22mammary+metastas%2A%22%5BTitle%5D%29%29+AND+%28%22Lymphedema%22%5BMeSH+Terms%5D+OR+%22lymphoedema%22%5BTitle%5D+OR+%22Lymphedema%22%5BTitle%5D+OR+%22lymphedemas%22%5BTitle%5D+OR+%22lymphatic+edema%22%5BTitle%5D+OR+%22oedema%22%5BTitle%5D+OR+%22edema%22%5BTitle%5D+OR+%22swelling%22%5BTitle%5D+OR+%22elephantias%2A%22%5BTitle%5D%29&sort=date&size=200&show_snippets=off) |

**Table1 Pubmed**

**Table2 Web of Science**

| #1 | **TI=((breast cancer) OR (breast tumor) OR (mammary neoplasm) OR (mammary carcinoma) OR (breast neoplasm) OR (breast carcinoma) OR (breast malignan*) OR (breast metastas*) OR (mammary malignan*) OR (mammary metastas*)）** | [74,122](https://www.webofscience.com/wos/alldb/summary/a185a14e-c582-49d5-8315-d6011533d3eb-0c6fcd3c/relevance/1) |
| --- | --- | --- |
| #2 | **TI=((Lymphedema) OR (lymphoedema) OR (Lymphedema) OR (lymphedemas) OR (lymphatic edema) OR (oedema) OR (edema) OR (swelling) OR (elephantias*) )** | [356,272](https://www.webofscience.com/wos/alldb/summary/9cb01f2c-a627-4e35-9810-3b4a852e8278-0c6fc1ff/relevance/1) |
| #3 | **TS=((Risk) OR (risk*) OR (risk factor) OR (risk factors) OR (age) OR (BMI) OR (modified radical mastectomy) OR (infection) OR (chemotherapy) OR (Radiotherapy) OR (physical activity) OR (exercise) OR (early edema) OR (seroma) OR (hypertension) OR (behavior) OR (prevention))** | [20,207,710](https://apps.webofknowledge.com/summary.do?product=UA&doc=1&qid=10&SID=7CVooLmJF7UM4rXMvx7&search_mode=AdvancedSearch&update_back2search_link_param=yes) |
| #4 | #1 AND #2 AND #3 AND | 1,113 |

**Table3 Embase**

| #1 | ('breast cancer '/exp OR 'breast cancer':ti OR 'breast tumor':ti OR 'mammary neoplasm':ti OR 'mammary carcinoma':ti OR 'breast neoplasm':ti OR 'breast carcinoma':ti OR 'breast malignan*':ti OR 'breast metastas*':ti OR 'mammary malignan*':ti OR 'mammary metastas*':ti) | 291,319； |
| --- | --- | --- |
| #2 | (lymphoedema/exp OR lymphoedema:ti OR lymphedema:ti OR lymphedemas:ti OR 'lymphatic edema':ti OR oedema:ti OR edema:ti OR swelling:ti OR elephantias*:ti) | 61,274 |
| #3 | (risk:ab,ti OR risk*:ab,ti OR 'risk factor':ab,ti OR 'risk factors':ab,ti OR age:ab,ti OR bmi:ab,ti OR 'modified radical mastectomy':ab,ti OR infection:ab,ti OR chemotherapy:ab,ti OR radiotherapy:ab,ti OR 'physical activity':ab,ti OR exercise:ab,ti OR 'early edema':ab,ti OR seroma:ab,ti OR hypertension:ab,ti OR behavior:ab,ti OR prevention:ab,ti) | 9664943 |
| #4 | #1 AND #2 AND #3 AND | 967 |

**Table4 Medline**

| #1 | TI ( (breast cancer) OR (breast tumor) OR (mammary neoplasm) OR (mammary carcinoma) OR (breast neoplasm) OR (breast carcinoma) OR (breast malignan*) OR (breast metastas*) OR (mammary malignan*) OR (mammary metastas*) ) AND TI ( (Lymphedema) OR (lymphoedema) OR (Lymphedema) OR (lymphedemas) OR (lymphatic edema) OR (oedema) OR (edema) OR (swelling) OR (elephantias*) ) AND AB ( (Risk) OR (risk*) OR (risk factor) OR (risk factors) OR (age) OR (BMI) OR (modified radical mastectomy) OR (infection) OR (chemotherapy) OR (Radiotherapy) OR (physical activity) OR (exercise) OR (early edema) OR (seroma) OR (hypertension) OR (behavior) OR (prevention) ) | 716 |
| --- | --- | --- |
